# Impact of Chronic Medical Conditions on Academics of Children in the Child Welfare System

**DOI:** 10.3389/fpubh.2018.00267

**Published:** 2018-09-20

**Authors:** Emily E. Whitgob, Irene Marilyn Loe

**Affiliations:** Stanford University, Stanford, CA, United States

**Keywords:** child welfare, academic achievement, chronic medical condition, school engagement, vulnerable population (source: MeSH, NLM)

## Abstract

**Objective:** Children in the Child Welfare System (CWS) are at high risk for multiple adverse outcomes. Since involvement in CWS and having a chronic medical condition are both risk factors for poor academic achievement, a logical view is that the combination is additive, increasing the odds of poor performance. However, several factors may complicate such an association. This study explores negative and positive factors that could affect academic achievement in children in CWS with chronic medical conditions.

**Method:** In a secondary data analysis of a nationally representative, longitudinal sample of children in CWS (*N* = 5,501), subjects were divided into three groups based on chronic medical condition: High Prevalence, Low Severity (HPLS; asthma, eczema, allergy, diabetes), Other (OTH; all other chronic conditions, including those with primary central nervous system involvement), and NONE (children with no chronic condition). Using weighted analyses, hierarchical logistic regression models addressed factors associated with academic achievement. Predictor variables included chronic condition group, sex, income level, case substantiation, home placement, and school engagement. Intelligence quotient was a covariate. Outcome variables were strong performances for reading and math, defined by standard score ≥85.

**Results:** In TOTAL group, 80% had strong reading; more in HPLS (85%) vs. NONE (79%) and OTH (80%), adjusted *F* = 433, *p* < 0.001. In TOTAL group, 67% had strong math; more in NONE (68%) and HPLS (68%) vs. OTH (60%), adjusted *F* = 1,278, *p* < 0.001. Models predicting to strong reading and math achievement were significant, *R*^2^ = 0.51, *p* < 0.001 and *R*^2^ = 0.43, *p* < 0.001, respectively. HPLS had increased odds of strong reading achievement (aOR 1.3, 95% CI 1.3–1.4); both HPLS and OTH had lower odds of strong math achievement (aOR 0.87 and 0.76), *p* < 0.001, respectively. Male sex had lower odds of strong reading (aOR 0.44) and math achievement (aOR 0.62); positive school engagement had higher odds of strong reading (aOR 1.18) and math achievement (aOR 2.04), all *p* < 0.001.

**Conclusion:** If true, our findings challenge the general belief that chronic illness can only be associated with negative outcomes and that cumulative adversities are simply additive in terms of risk. Increased contact with the medical system may provide an opportunity for improving reading achievement for children in CWS and promoting positive school engagement.

## Introduction

Children in the Child Welfare System (CWS) are vulnerable by definition, as they are referred to the system for suspected abuse and/or neglect. Whether or not the claim of maltreatment is substantiated, children in CWS are at high risk for multiple adverse outcomes. Involvement in CWS has been associated with worse academic outcomes than no involvement (1). Although there are resilience factors that moderate the negative effects of childhood adversity on academic scores, children who are chronically maltreated have lower standardized test scores than children who are not chronically maltreated (2). A study of 6–10 year old children with maltreatment found lower math and reading achievement scores compared to population norms, with greater effects for math (approximately one-third to almost one SD lower scores) compared to reading (less than one-third SD) (2). In a longitudinal study, early abuse and neglect during the first 5 years of life was consistently associated with more interpersonal problems and lower academic achievement from childhood through adulthood at 32–34 years of age (3).

In addition to adverse academic outcomes, there are adverse outcomes for multiple domains of function, including social, behavioral, emotional, and developmental concerns, in addition to the physical health of children in CWS. A nationally representative, longitudinal study of children in CWS by Ringeisen et al. (4) found that approximately 50% of children were identified as having special health care needs (SHCNs) across 3 years of follow-up, significantly higher than an estimate of 13–19% of children in the US population with SHCNs. Children and youth with SHCNs are defined as “those who have or are at risk for a chronic physical, developmental, behavioral, or emotional condition and who also require health and related services of a type or amount beyond that required by children generally” (5). Developmental, behavioral, and emotional outcomes of children in CWS have been more heavily studied (6, 7) than physical health, despite findings that children in CWS have a rate of chronic medical conditions that is more than 1.5 times the general population (4, 8). Asthma is the most prevalent chronic health condition in CWS (4). The presence of adverse childhood experiences (ACEs) in children in CWS is associated with increased risk of chronic medical conditions. A study of young children age 18–71 months in CWS found that almost all children (98.1%) were reported to have at least one ACE during the lifetime and an average of 3.6 ACEs. In this study, each ACE increased the odds of having a chronic medical condition by 21% (9).

Childhood chronic illness apart from CWS is an independent risk factor associated with worse academic outcomes in children compared to those without (10). The presence of health adversities has been associated with poor academic outcomes with a dose-dependent effect (11). In a longitudinal study of 8 and 9 year old children, there were incremental decreases in academic achievement scores with each additional health adversity found both cross-sectionally at ages 8–9 years and longitudinally at ages 10–11 years (11).

Consistent with the concept of cumulative risk, it might follow that children who are both in CWS and also have a chronic illness would have cumulative effects resulting in even worse academic outcomes. Cumulative risk is a framework to view the context in which a child grows (12). It builds on Bronfenbrenner's ecobiological model, a theory of understanding human development through interactions between genetic and environmental factors. In contrast with a single risk factor, exposure to multiple risk factors puts children at increased odds for maladaptive outcomes in all areas of development (12).

To our knowledge, this exploratory study is the first to evaluate academic achievement of children in CWS who have chronic medical conditions. Since involvement in CWS and the presence of a chronic medical condition are both risk factors for poor academic achievement, a logical view would be that the combination is simply additive, increasing the odds of poor performance. However, several factors may complicate such an association.

Children with chronic medical conditions require frequent medical contact, yet these children who are also involved in CWS often have limited access to medical homes (13). The medical home has been defined as “an approach to providing comprehensive and high quality primary care” that has several features, including being accessible, family-centered, continuous, comprehensive, coordinated, compassionate, and culturally effective (11). The medical home “builds partnerships with clinical specialists, families, and community resources” (14). For children in CWS, their contact within primary or subspecialty care ranges from regular to fragmented or intermittent. Frequent medical contact may confer benefits by increasing the likelihood of surveillance, detection of developmental problems and learning difficulties, and subsequent referral for school evaluation. Ideally an ongoing relationship with a trusted adult may provide mentorship and positive role modeling. For this population of children where a medical home is not easy to come by, every contact with a medical provider is even more crucial in terms of observing the child's development and intervening when appropriate. Using a lifecourse health development model described by Halfon et al. (15), participation in CWS and having a chronic illness in childhood are both risk factors for poor academic achievement and an overall lowered trajectory across the lifecourse for multiple outcomes. Quality health services might serve as a positive factor and, for the population in this study, the opportunity for frequent contact with these quality services may move children toward a “healthy” trajectory (15).

In addition to quality health services and frequency of medical contact, positive experiences with school could also positively influence academic achievement. School engagement has been defined as the level of participation, interest, and effort toward school and has been studied as a means of combating declining academic motivation and achievement (16). School engagement is a multifaceted construct that can be divided into three dimensions–behavioral, emotional, and cognitive engagement. Behavioral engagement refers to participation or involvement in academic and social or extracurricular activities; it is considered crucial for achieving positive academic outcomes and preventing dropout. Emotional engagement refers to positive and negative reactions to teachers, classmates, academics, and school; it presumably creates ties to school and influences student willingness to do academic work. Cognitive engagement generally refers to investment—thoughtfulness and willingness to exert the required effort to understand complex ideas and master difficult skills (16). Given the complexity of the interactions between a child and various school experiences, there are multiple ways in which the adults in a child's life could also influence the child's school engagement, ranging from physically providing opportunities to participate in extracurricular activities to conveying that effort toward academics is important and providing children with encouragement and motivation.

In this study we were interested in exploring both negative and positive factors that could affect academic achievement in children in CWS with chronic medical conditions. Based on the literature documenting independent associations of CWS involvement and chronic medical conditions with adverse academic outcomes, we suspected that the presence of both would adversely affect academic outcomes. However, we were also interested in whether other factors related to chronic illness and school engagement might be positive predictors of stronger academic achievement in this high-risk population. Since the longitudinal dataset used for the study did not include a comparison control group of children outside CWS, we categorized children with chronic medical conditions into subgroups to better understand the potential impact of chronic conditions. Children were divided into the following subgroups: (1) high prevalence, low severity conditions group (HPLS) consisting of eczema, allergies, asthma, and diabetes, that often bring children into frequent medical contact but do not have primary central nervous system (CNS) involvement; (2) other chronic medical conditions (other or OTH), including those with primary CNS involvement (i.e., brain tumor, cerebral palsy, epilepsy/fits/convulsions) and (3) no chronic medical condition (NONE). We hypothesized that: (1) OTH group would have poorer academic achievement in both reading and math compared to the NONE and HPLS groups due to higher severity illness and CNS involvement; (2) HPLS group would have intermediate academic achievement in both reading and math–better than OTH group but below the NONE group; and (3) NONE group would have the strongest academic achievement of the three groups. Using a definition of school engagement as the level of participation, interest, and effort toward school (16), we also hypothesized that positive school engagement would be associated with stronger academic scores and serve as a positive predictor.

## Methods

The National Survey of Child and Adolescent Well-Being (NSCAW I) is the first nationally representative, longitudinal study of children age < 15 years (*N* = 5,501) who had contact with CWS during a 15-months period starting in October 1999. These children had investigations by Child Protective Services (CPS) for abuse or neglect. Notably, involvement in CWS includes any case with an allegation of maltreatment; the case may not have been substantiated, and the child often stayed in the original home but may have had short-term out-of-home/foster placement during the follow-up period. The NSCAW used a national probability sampling strategy to select a total of 100 counties representing CWS agencies. Child-level data was obtained for 92 counties over four subsequent waves of data collection (12-, 18-, 36-, and 59–97 months) after baseline. Eight counties were ineligible due to state laws requiring first contact of potential participants by CWS rather than NSCAW study personnel (17). Families were invited to participate prospectively, prior to collection of outcome and care disposition data. Both the national representation of children in CWS and longitudinal collection period were among the specific reasons this dataset was selected.

In addition to its longitudinal design and national representation, the NSCAW dataset offers rich information from multiple sources of information on children's health and academics. Data were collected from caregiver report, caseworker and teacher report, and direct measurement or interview with children. Fortunately, the child's disposition toward learning and school, one of our positive predictors of interest, was assessed through a child interview instrument on school engagement. We were therefore able to go beyond the typical measures of academic achievement with this assessment of child attitudes toward learning and school. Unfortunately, the dataset was limited by not including severity data for children's chronic conditions, nor did it provide information on number of contacts with a physician.

We conducted secondary data analysis of Waves 1 (baseline) and 4 (36 months post-baseline). Children included from Wave 1 data needed to be at least 36 months old at Wave 1 in order to meet age eligibility (72 months) for the measure of academic achievement at Wave 4. Wave 4 was chosen as the endpoint because we wanted the longest timeframe possible with the greatest number of respondents. There were fewer responses in Wave 5 due to attrition. Furthermore, the academic tests used at Wave 5 were different from those used in prior waves, making scores less comparable. Demographics and medical conditions were gathered from Wave 1 data. Child academic achievement scores and school engagement data were gathered at Wave 4. The NSCAW study was approved by the IRB of the Research Triangle Institute (RTI), a not-for-profit organization that conducts research for national, state, and local government agencies, that is incorporated by the University of North Carolina at Chapel Hill, Duke University in Durham, and North Carolina State University in Raleigh. RTI worked with the Administration for Children and Families within the Department of Health and Human Services and with a team of collaborators in the design, execution, and overall coordination of the NSCAW study. Caregivers or the person or agency with legal guardianship provided consent for participation in the study; children age 7 and older provided assent. Our use of secondary data resulted in the study being deemed exempt by the Institutional Review Board at the authors' university because the data were deidentified.

## Measures

### Demographics and participant characteristics

Baseline demographic characteristics are described in Table 1. There were statistically significant differences across the three conditions for all demographic variables, therefore selected variables were included in the regression models predicting to academic achievement. Child age, sex, race, and ethnicity were obtained from caregiver interview. Household poverty status was determined by the child's family income and number of adults and children in the household per US Census Bureau guidelines. Poverty was dichotomized as above or below the county poverty level. The categorical description of income was used for consistency with other demographic variables that were also categorical. Substantiation describes the CWS decision about the validity of maltreatment allegations. Cases that were not substantiated may still have had evidence of maltreatment but not enough to confirm the allegation. Similar to another study using NSCAW I data and the criteria used by the annual national reports on child maltreatment based on the National Child Abuse and Neglect Data System (18), maltreatment was stratified by a case being substantiated or unsubstantiated, which was a proxy for severity of maltreatment. Given that placement instability has been linked with increased emotional dysregulation and subsequent lower academic achievement in another NSCAW I study (19), home placement was included in our models. Living arrangements at Wave 4 were described as either living in the home with parent(s) or out of the home. Children in foster care >1 year at the time of Wave 1 data are in a separate dataset; however, children in the current dataset may have had temporary placement (defined as < 1 year) in foster care between Waves 1 and 4.

**Table 1:** Participant characteristics by chronic condition group.

**Characteristics**	**Total**		**Prevalence, % (SE)**		
	***n***	**% (SE)**	**HPLS**	**OTH**	**NONE**
**AGE (YEARS)**					
0–2	1,763	18.9 (0.014)	28 (0.152)	14 (0.081)	19 (0.030)
3–5	717	20.3 (0.015)	24 (0.051)	19 (0.021)	20 (0.021)
6–10	1,272	36.2 (0.027)	29 (0.074)	38 (0.046)	37 (0.038)
≥11	1,029	24.6 (0.055)	19 (0.042)	29 (0.045)	25 (0.074)
**SEX**					
Male	2,355	49.8 (0.037)	60 (0.131)	47 (0.053)	49 (0.052)
Female	2,427	50.2 (0.037)	40 (0.131)	53 (0.053)	51 (0.052)
**RACE AND ETHNICITY**					
Black, non-Hispanic	1,557	27.8 (0.046)	29 (0.067)	20 (0.022)	29 (0.061)
White, non-Hispanic	2,063	47.0 (0.035)	54 (0.117)	59 (0.052)	44 (0.046)
Hispanic	819	18.3 (0.035)	12 (0.188)	16 (0.053)	20 (0.052)
Other	328	6.9 (0.005)	5 (0.021)	5 (0.006)	8 (0.008)
**HOUSEHOLD POVERTY STATUS**					
Above poverty level	2,041	43.7 (0.035)	48 (0.105)	44 (0.059)	43 (0.045)
Below poverty level	2,741	56.3 (0.035)	52 (0.105)	56 (0.059)	57 (0.045)
**PLACEMENT**					
Out of home	805	9.8 (0.136)	8 (0.193)	9 (0.009)	10 (0.177)
In the home	3,964	90.2 (0.136)	92 (0.193)	92 (0.009)	90 (0.177)
**SUBSTANTIATION**					
No	1,378	67.7 (0.043)	65 (0.173)	66 (0.095)	69 (0.062)
Yes	2,381	32.3 (0.043)	35 (0.173)	35 (0.095)	32 (0.062)
**SCHOOL ENGAGEMENT**					
Positive	1,532	60.9 (0.260)	60 (0.056)	55 (0.036)	62 (0.344)

*All of the percentages are weighted; numbers are unweighted*.

*SE, standard error; HPLS, high prevalence, low severity; OTH, other*.

### Chronic health conditions

Chronic health condition status was the primary predictor variable. Caregivers reported on 27 chronic health conditions from a non-categorized list of diagnoses of varying severity and specificity (4). Caregivers were asked whether the child had any health problems that “last a long time or come back again and again.” Mental health conditions were not included in this list and are described elsewhere in the NSCAW; they were not analyzed here given our focus on chronic physical health conditions. Children were divided into three groups based on the diagnoses caregivers confirmed: high prevalence, low severity (HPLS) included asthma, eczema, severe allergy, and diabetes; all other chronic conditions (OTH); or no chronic condition (NONE). The HPLS category was deliberately designed to include chronic conditions that likely require frequent medical contact and were unlikely to have direct effects on the central nervous system (CNS) that could lead to significant impairments in learning. The asthma category occurred at similar rates in the NSCAW as those documented nationally (approximately 8%) (5). Other atopic illnesses were grouped with the HPLS category, including eczema and allergies, defined as severe allergies in the NSCAW list of conditions. Eczema and allergies occurred at rates of approximately 2–4% (5). Therefore, the HPLS category included conditions with specificity (i.e., diabetes) and high occurrence in the form of asthma and atopic disease. In contrast, the OTH category included conditions with primary CNS involvement (i.e., seizures, traumatic brain injury, brain tumor), potential serious CNS involvement (i.e., emboli and stroke associated with sickle cell anemia), and conditions that were not well-defined or of low prevalence (i.e., other birth defect, physical deformities). If a HPLS condition co-occurred with a diagnosis from the OTH group, then the child was classified in the OTH group.

### School engagement

Children's “school engagement” was included as a predictor variable. The NSCAW asked all children age 6 and older who were attending school to answer a series of 11 questions about their involvement in school, including relationships with peers and teachers, academic assignments, and classroom behavior. The questions were adapted for the NSCAW study from the national Outcomes of the Drug-Free Schools and Communities Act (DFSCA), sponsored by the US Department of Education (20). In the DFSCA programs, the questions were administered to 10,000 fifth and sixth graders from 19 school districts across the US (20). For the NSCAW, factor analysis was used to create the school engagement scale; items show high internal consistency (α = 0.84) with higher scores indicating higher school engagement (21). The questions, rated on a 4-point Likert scale, were administered through child interview. The self-report nature of this tool is a strength, as many studies focus on academic achievement through test scores alone. Examples of interview questions include: “How often do you enjoy being in school? How often do you try to do your best in school? How often do you get your homework done?” If a child answered in a positive manner to at least half of the questions, then it was coded as positive school engagement. Responses were considered positive if the child answered “often” or “almost always” for questions worded in the positive direction (i.e., “How often do you enjoy being in school?”) or “never” or “sometimes” for questions worded in the negative direction (i.e., “How often do you hate being in school?”) In this exploratory study of positive and negative factors affecting academic outcomes, we sought to learn if positive school engagement would be associated with strong academic scores and serve as a positive predictive factor.

### Academic achievement measures

Children completed the reading and math subtests from the Woodcock-McGrew-Werder Mini-Battery of Achievement, a standardized measure of academic skills for children 6 years and older (22). Reading and math subtest scores served as the primary academic achievement outcome measures. Scores have a mean of 100 and standard deviation (SD) of 15. Results were dichotomized as strong vs. weak performance if the standard score was ≥85 (within one standard deviation of the mean or higher) and weak performance if the standard score was < 85. Categorical scores were used for academic measures, which maintained consistency with all other dichotomous outcomes. Scores within the average range demonstrated sufficient academic performance for the purposes of this study. The Kaufmann Brief Intelligence Test (KBIT), a brief, individually administered measure of verbal and non-verbal intelligence has a mean of 100, SD 15 (23). KBIT scores were included in the models as a covariate to control for children's intelligence level.

## Analyses

Group differences in demographic variables, school engagement, academic achievement and cognitive scores were analyzed with weighted *X*^2^ and weighted ANOVA. Using weighted data in order to account for the complex sampling design of NSCAW, separate hierarchical logistic regression models were conducted to evaluate the strength of associations among various demographic factors, chronic illness category, and school engagement with strong academic performance for reading and math. Predictors in the model included the following: sex, poverty level, living arrangements (in- vs. out-of-home) and CWS case substantiation in Step 1; chronic condition status in Step 2; and school engagement in Step 3. KBIT score was included as a covariate. Complex samples commands in SPSS Statistics 24 were used to account for weighting in the dataset. The reference group for all analyses consisted of children in NSCAW who did not carry a diagnosis of a chronic condition, those in the NONE category. For each of the categorical demographic variables we selected reference categories as follows: female, living in the home, above poverty level, and unsubstantiated case status. Traditionally these groups have better academic outcomes. We used a significance level of *p* < 0.001 given the large sample size of this weighted, nationally representative sample.

We chose to dichotomize performance into average/above average scores vs. below average scores instead of analyzing score as a continuous variable. In this way, we intended to identify the proportion of children who were likely to need special services in academic areas. Using a broad perspective to understand categorical academic performance of children with chronic conditions who are in CWS is more meaningful for clinical practice recommendations than a granular interpretation of individual scores.

## Results

From the total sample of *N* = 5,501 children, 2,726 (50%) had complete data for the primary outcomes of reading and math academic achievement scores and demographic variables; they were included in the weighted analyses. The decreased sample size reflects that children had to be at least 72 months of age at Wave 4 in order to be age eligible for the academic achievement measures; infants and children < 3 years of age at the time of enrollment at Wave 1 were not eligible for the primary outcomes measures at Wave 4. Mean scores for each assessment by chronic condition group are described in Table 2. Weighted ANCOVA, covarying for intelligence level, showed statistically significant differences across chronic condition groups although scores were in the average range. The lowest mean scores were the math achievement scores for children in the OTH group, which were significantly lower than both the HPLS and NONE groups, *p* < 0.001. The three groups (HPLS, OTH, NONE) had mean reading and math standard scores in the average range (i.e., 88–96), although scores were below the population mean of 100.

**Table 2:** Mean IQ and academic achievement scores by chronic condition group.

**Test**	**Total**	**HPLS**	**OTH**	**NONE**
KBIT composite normalized score (SE)	95 (0.01)	96 (0.01)	94 (0.01)	95 (0.02)
WJ reading standard score (SE)	97 (0.03)	97 (0.02)	96 (0.03)	98 (0.04)
WJ math standard score (SE)	93 (0.03)	93 (0.01)	88 (0.01)	93 (0.04)

*HPLS, high prevalence, low severity; OTH, other; KBIT, Kaufman Brief Intelligence Test; WJ, Woodcock Johnson; SE, standard error*.

Weighted *X*^2^ tests showed that the percentage of children with strong reading scores is significantly higher in the HPLS group (85%), compared to the NONE (79%) and OTH (80%) groups, adjusted *F* = 433, *p* < 0.001. The percentage of children with strong math scores was the same for the HPLS (68%) and NONE (68%) groups. The OTH group (60%) was significantly lower; adjusted *F* = 1,278, *p* < 0.001.

Weighted hierarchical logistic regression models predicting to strong reading and math achievement are shown in Tables 3, 4, respectively. The overall model predicting to strong reading achievement was significant with Nagelkerke *R*^2^ = 0.51, *p* < 0.001. At each step, the overall *R*^2^ was relatively unchanged with shifts noted in the odds ratios and significance levels of the demographic variables. The HPLS group had an increased adjusted odds ratio of strong reading achievement. The overall model predicting to strong math achievement was significant with Nagelkerke *R*^2^ = 0.43, *p* < 0.001. Similar to reading, at each step the model *R*^2^ was relatively unchanged. Both HPLS and OTH groups had decreased adjusted odds of strong math achievement. Male sex was associated with significantly lower adjusted odds of strong reading and math achievement while positive school engagement was associated with higher adjusted odds of strong reading and math achievement. We further explored the possibility of a chronic condition group status by school engagement interaction by placing this interaction term in the logistic regression model; however, there was collinearity among variables, rendering the model unreliable.

**Table 3:** Logistic regression model predicting to strong reading scores.

	**Step 1**			**Step 2**			**Step 3**		
	***R***^**2**^ = **0.51**, ***p***<**0.001**			***R***^**2**^ = **0.51**, ***p***<**0.001**			***R***^**2**^ = **0.51**, ***p***<**0.001**		
**Predictors**	**aOR**	**95% CI**	***p***	**aOR**	**95% CI**	***p***	**aOR**	**95% CI**	***p***
^*^Male sex	**0.47**	0.44–0.51	< 0.001	**0.47**	0.43–0.50	< 0.001	**0.44**	0.40–0.47	< 0.001
Out of home	1.07	0.79–1.43	0.656	1.07	0.79–1.43	0.652	1.00	0.71–1.41	0.985
Poverty	1.00	0.93–1.07	0.938	1.01	0.94–1.08	0.832	0.97	0.90–1.05	0.408
Substantiated	1.06	1.00–1.13	0.056	1.06	1.00–1.13	0.065	1.11	1.02–1.20	0.016
^*^HPLS				**1.24**	1.20–1.28	< 0.001	**1.31**	1.26–1.36	< 0.001
OTH				0.94	0.91–0.98	0.003	0.98	0.93–1.02	0.290
^*^Positive school engagement							**1.18**	1.09–1.28	< 0.001
**COVARIATE**									
^*^K-BIT	1.1	1.16–1.17	< 0.001	1.16	1.16–1.17	< 0.001	1.17	1.16–1.17	< 0.001

*OR indicates odds ratio; CI, confidence interval; HPLS, high prevalence, low severity; OTH, other; KBIT, Kaufmann Brief Intelligence Test*.

* *Statistically significant values with p < 0.001; significant aOR are in bold*.

**Table 4:** Logistic regression model predicting to strong math scores.

	**Step 1**			**Step 2**			**Step 3**		
	***R***^**2**^ = **0.42**, ***p***<**0.001**			***R***^**2**^ = **0.43**, ***p***<**0.001**			***R***^**2**^ = **0.43**, ***p***<**0.001**		
**Predictors**	**aOR**	**95% CI**	***p***	**aOR**	**95% CI**	***p***	**aOR**	**95% CI**	***p***
^*^Male sex	**0.62**	0.59–0.66	< 0.001	**0.62**	0.56–0.67	< 0.001	**0.62**	0.58–0.66	< 0.001
Out of home	1.12	0.88–1.43	0.325	1.10	0.87–1.39	0.412	1.03	0.78–1.35	0.827
Poverty	1.04	0.99–1.09	0.112	1.05	1.00–1.10	0.036	1.09	1.03–1.16	0.007
Substantiated	0.96	0.91–1.01	0.077	0.96	0.91–1.01	0.093	1.01	0.94–1.08	0.845
^*^HPLS				**0.89**	0.87–0.92	< 0.001	**0.87**	0.85–0.90	< 0.001
^*^OTH				**0.68**	0.67–0.70	< 0.001	**0.76**	0.74–0.79	< 0.001
^*^Positive school engagement							**2.04**	1.91–2.18	< 0.001
**COVARIATE**									
^*^K-BIT	1.13	1.12–1.13	< 0.001	1.13	1.12–1.13	< 0.001	1.13	1.12–1.13	< 0.001

*OR indicates odds ratio; CI, confidence interval; HPLS, high prevalence, low severity; OTH, other; KBIT, Kaufmann Brief Intelligence Test*.

* *Statistically significant values with p < 0.001; significant aOR are in bold*.

## Discussion

In summary, in this study of a large, nationally representative sample of US children in CWS, we found that children in CWS with high prevalence, low severity chronic conditions without CNS involvement (HPLS) have a higher odds ratio for strong reading scores in comparison with children with chronic conditions that include CNS involvement (OTH), consistent with our hypothesis. However, HPLS also had higher odds compared to children without chronic conditions (NONE), whereas the OTH group had almost even odds (OR 0.98) with the NONE group. Consistent with our hypothesis, the HPLS group had lower odds ratio for strong math scores compared to the NONE group and higher odds ratio compared to the OTH group. Positive school engagement was associated with significantly higher adjusted odds ratio for strong reading and math scores, although the elevated odds for math is at a more clinically relevant level than the odds for reading (aOR of 2.04 vs. 1.18). The results support past findings that chronic medical conditions often are independently associated with lower reading and math achievement, but this association may not hold in a subset of socially vulnerable children.

There are several possible explanations for these findings. Children in CWS who normally lack adult champions may have had greater access to adult champions (e.g., medical providers) if they had a chronic medical condition. Perhaps for children in CWS, this increased positive contact with an adult champion may counterbalance other negative environmental risk factors (i.e., abuse and neglect). This is supported by some prior studies of children not in CWS that found an association between better academic performance and specific chronic medical conditions (e.g., asthma and obesity) (10). The conditions of interest in this study included asthma. Another possible explanation is that the selected chronic conditions without CNS involvement may have provided an opportunity for the child in CWS to have additional contact with interested health care providers and other adults. This contact may counterbalance environmental risk factors of involvement in CWS. Schultz et al propose that teachers, coaches, counselors, clergy or neighbors may act as positive factors at the community level (24). If this explanation is correct, then medical clinicians may also belong to this protective network. The cumulative effects of child welfare involvement and having a chronic condition did not manifest as poor reading scores. Although we did not find a positive association between the HPLS group and math scores, the odds of strong math scores for the HPLS were no worse than the OTH group.

The differences in the odds of strong reading compared to strong math scores is in line with previous research on standardized test scores of children who have been maltreated that found larger differences in math (i.e., lower scores) compared to reading (2). The greater effect on math might indicate the need for more attention or a different approach to remediation. Why is there a positive association with reading and not with math? One possible explanation pertains to differences in how the two subjects are taught. With reading, once reading skills such as decoding, are taught and mastered, learning might be continuous, with additional assignments in other subjects, such as social studies and science, refining and strengthening reading skills. Math concepts and skills are taught in discrete lessons and require the mastery of discrete skills or operations. If a child misses 2 weeks of school due to illness or chaos during a CWS investigation, it might be more difficult to acquire the missed math material.

We also found that positive school engagement was associated with higher odds of strong reading or math achievement. This finding is consistent with other studies that found associations between poorer student engagement and lower academic achievement in children with special healthcare needs (10). Medical providers are in a position to inquire about children's attitudes about school, monitor performance, refer for evaluations and services, and inform parents about educational rights and how to best support the child to promote positive school engagement. Given that positive school engagement is a positive predictor of school achievement, then promoting that engagement is another way that healthcare professionals can contribute to improving academic outcomes. Further study can provide a practical understanding of the provider's role in this regard.

This study had several limitations due to the design of the dataset. There is no comparison control group outside of the CWS. We compare our population of children within CWS based on subcategories of chronic medical condition and those without. While diabetes may have some CNS effects, it was a condition included by NSCAW that was likely to have frequent medical contact so it was included with the HPLS group; it had an overall low occurrence (< 1%) in the study. There was no direct measure of illness severity or direct measure of frequency of medical contact. The OTH group was heterogeneous and included conditions that might bring children into frequent medical contact. However, given the low prevalence of several of the conditions, the further subdivision of conditions into additional subcategories would have reduced power to detect associations. In addition, results with several additional subcategories would be more difficult to interpret relative to the NONE condition. The study was designed to be nationally representative of children in CWS, but it was not designed to be nationally representative of chronic medical conditions. Caregivers were asked to report on health problems that “last a long time or come back again and again,” but are subject to recall bias and may have over- or under- reported chronic conditions. The NSCAW dataset did not include a description of the clinical services children received; some clinical programs may include contact with social workers, dietitians, and child life specialists, all of whom may provide support for children toward a healthy lifecourse trajectory (15).

The gaps and limitations in our study can be used to guide the design of future studies on this topic. For example, collaboration with researchers from other countries that have centralized systems of care and comprehensive data on child health would add rich detail to the understanding of the academic outcomes of children who have been part of the child welfare system and also have chronic medical conditions. We were also unable to assess interactions between positive school engagement and chronic condition subcategory. In the future, the malleability of school engagement could be studied with potential intervention targets in mind. Future prospective studies could also focus on interventions targeting primary care providers and systematic study of the medical home and care coordination in this vulnerable population.

## Conclusion

If true, the findings from our exploratory study challenge the general belief that chronic illness can only be associated with negative outcomes and that cumulative adversities are simply additive in terms of risk. Better understanding of what buffers the adverse impact of early-life events remains an important goal, particularly in the CWS population. This understanding may help reduce the negative, often cumulative, educational impacts of child maltreatment (25) and move children toward a healthy trajectory over the lifecourse. Increased contact with the medical system may provide an opportunity for improving reading achievement for children in CWS and promoting positive school engagement. Medical providers are in a position to monitor academic concerns, make referrals, and influence academic achievement, which could prevent secondary complications of child maltreatment. We recommend this involvement in the lives of children in CWS who have chronic conditions be further studied to measure its impact on this vulnerable group's academic performance.

## Author contributions

EW conceptualized and designed the study, participated in data analysis, drafted the initial manuscript, and reviewed and revised the manuscript. IL participated in conceptualization and study design, supervised data analysis, and provided review and revision of the manuscript. All authors approved the final manuscript as submitted and agree to be accountable for all aspects of the work.

### Conflict of interest statement

The authors declare that the research was conducted in the absence of any commercial or financial relationships that could be construed as a potential conflict of interest.
